# The second chance story of HIV-1 DNA: Unintegrated? Not a problem!

**DOI:** 10.1186/1742-4690-5-61

**Published:** 2008-07-09

**Authors:** Yuntao Wu

**Affiliations:** 1 Department of Molecular and Microbiology, George Mason University, Manassas, VA, 20110, USA

## Abstract

Accumulation of high levels of unintegrated viral DNA is a common feature of retroviral infection. It was recently discovered that coinfection of cells with integrated and unintegrated HIV-1 can result in complementation, allowing viral replication in the absence of integration. This new mode of HIV-1 replication has numerous implications for the function of unintegrated viral DNA and its application as a therapeutic vector.

## Introduction

With retroviruses such as HIV, life seems to be simple and straightforward. As a single infectious particle, the virus converts its RNA genome into DNA and then incorporates it into the host genome. Once this happens, the rest of the viral life cycle is largely a happy free ride from the host. However, for the viral population as a whole, the truth is that only a very small proportion of the viruses have such a productive life. The vast majority of the viral DNA remains isolated from the host chromatin [1-8]. These DNA molecules are euphemistically referred to as the "unintegrated"; in reality, they are the "left behind" and down regulated (gene expression is low and restricted to only early genes [9-11]). The stakes are high; they are at risk of being destroyed and cleared [12,13]. We still do not understand why most HIV DNA cannot or does not integrate, and other questions remain as well: is there something wrong with these "unintegrated," and do they deserve a second chance?

Answering these questions is not as simple as it seems. First, within a viral population, we do not know which viral DNA is destined to integrate, and there is no marker to differentiate this phenotype. Second, against a background of viral activities from both the integrated and the unintegrated, it is difficult to monitor and track viral behavior from the unintegrated alone. In spite of these hurdles, in the recent article by Gelderblom and co-authors [14], these questions were elegantly addressed using a very creative approach. The authors employed coinfection of cells with the wild-type virus and an integrase mutant, both of which were labelled with different fluorescent reporters. This permitted tracking and delicate differentiation of the wild-type and the unintegrated viruses.

To address the question of whether the unintegrated viral DNA remains functional, the authors used an integrase inhibitor and an integrase mutant virus, D116N [15]. They also tagged the viral early genes with green fluorescent protein (GFP) and a late gene with murine Heat Stable Antigen (HSA). When cells were infected with D116N, or with the wild-type virus in the presence of the integrase inhibitor, approximately 25% of the cells expressed low levels of viral genes from the unintegrated DNA, in comparison with cells infected with only the wild-type virus. The authors also found that 96% of the D116N-infected, GFP^+ ^cells expressed only the early genes. These results are consistent with previous findings that unintegrated virus can transcribe both the early (multiply spliced) and late (partially spliced and unspliced) genes, but only the early genes are measurably translated due to a lack of sufficient Rev function [9-11].

Remarkably, when the DsRedX-labelled wild-type virus was used to coinfect with the GFP-labelled D116N, the authors were able to demonstrate that the wild-type virus can chase a large amount of unintegrated HIV DNA into active templates through the stimulatory effect of Tat. Additionally, the wild-type virus can even drive the unintegrated viral DNA to express late genes through the action of Rev. Furthermore, the RNA genome transcribed from the unintegrated DNA can be packaged into the virion and is able to effectively compete with the wild-type genome for packaging. These results clearly suggest that the unintegrated DNA molecules have the full potential in this regard of any HIV DNA. Their limitations in expressing viral genes appear to be only temporary, imposed by the lack of sufficient Tat and Rev function.

With this understanding of their full potential, the next question of whether these unintegrated DNA molecules deserve a second chance becomes obvious. Yes, they do! Indeed, the authors confirmed that in the presence of the second virus, the unintegrated HIV DNA molecules were driven to express both early and late genes, as well as viral genomes that were subsequently packaged and released from the cell. They thus started on a new journey that gave them a second opportunity to integrate. As the authors concluded, this complementation between the few integrated and the majority unintegrated would prevent possible losses of viral genetic diversity.

## Discussion

**Figure 1:**
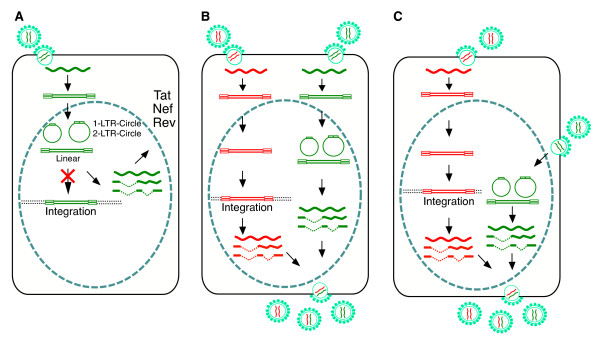
**Model of complementation between unintegrated and integrated HIV-1**. (A) Viral transcription in the absence of integration generates all classes of viral transcripts, but only early proteins such as Tat, Rev, and Nef are synthesized at low levels. Tat and Nef can modulate cellular conditions. Viral replication does not occur without integration, but infection by a second virus can rescue the unintegrated viral genomes. (B) Coinfection of a cell by multiple particles can lead to accumulation of unintegrated viral DNA. However, an integrated provirus can rescue the genomes of the unintegrated viral DNA, preventing possible losses of viral genetic diversity. (C) Superinfection of a productively infected cell may not require new integration of the incoming virus, thus reducing steps required for viral replication and avoiding excessive integration to disrupt cellular function.

Extrapolating from this modelling study, we can imagine three different scenarios in which the unintegrated viral DNA might contribute to a productive viral replication cycle. As shown in Figure 1A, during primary infection, in some situations where integration is restricted, because of either cellular restrictions or unknown viral processes, the unintegrated HIV DNA can still synthesize low levels of early proteins such as Tat, Rev, and Nef [9-11,16,17]. Both Tat and Nef can modulate T cell activity to facilitate activation [9,18-21]. In particular, Nef does not increase the extent of T cell activation; it only increases the number of T cells that can be activated [9,19-21]. This would expand cellular targets for viral infection, since a lot more cells are available for productive viral replication. In resting T cells cultured in vitro, viral DNA synthesis maximizes at around 2 days post infection, and the unintegrated viral DNA has a half life of about 1 to 2 days [12,13]. Some of the viral DNA may remain rescuable for weeks, since productive viral replication can be initiated with T cell activation [9,13,22-26]. In human macrophages, the unintegrated viral DNA can persist for as long as 30 days [11]. It is unlikely that the unintegrated DNA can still integrate after a certain time when the preintegration complex is disassembled. Nevertheless, the unintegrated DNA may still be rescuable by a second virus. In this sense, the unintegrated viral DNA would also constitute a viral reservoir. Certainly, the presence of such unintegrated reservoir has been detected in most of untreated HIV patients and in some of the infected patients on highly active antiretroviral therapy (HAART) [27]. This unintegrated reservoir is labile, but is inducible and functional even in some HAART-treated patients [27].

The unintegrated viral DNA molecules do not simply wait for the rescuer; they also actively synthesize early proteins such as Tat and Nef to modulate cellular conditions. To be rescued, the unintegrated DNA has to meet several conditions. First, its low-level viral activity should not kill the cell carrying the unintegrated viral DNA. Second, it should prime a cellular condition that favors the second virus after the initial integration attempt fails. This would ensure that the rescuer would not be trapped in the same situation. Third, although perfect fitness is not required, any rescuable virus should have a selective advantage equal or better than that of the rescuer, in terms of the ability to compete for packaging and promoting favorable cellular conditions. One remaining issue for this scenario, however, is whether the unintegrated virus may prevent secondary infection. Although Nef expressed from unintegrated DNA can also down-modulate CD4 [17], it is unlikely that the down-modulation can reach such a severity that it completely prevents superinfection [28].

In the second scenario (Figure 1B), where local virus concentrations are high, multiple coinfection of a cell, such as the infection of cells in lymphoid tissues, may occur. In this case, not every virus can integrate, and if some viruses fail, an integrated virus within the same cell would be able to rescue and complement the unintegrated viruses, preventing possible dwindling of the viral genetic repertoire [14]. HIV coinfection is also an important source of viral recombination which may increase the fitness of the virus [29]. Coinfection is certainly detected frequently in patients and is known to contribute to viral genetic diversity [29].

In the third scenario (Figure 1C), it is also possible that superinfection of an already productively infected cell may not always require new integration for the incoming virus. The incoming HIV DNA could be quickly used to express viral genes and be assembled into virion particles, with the help of Tat, Rev, and the assembly factors from the integrated provirus. This would facilitate viral replication and avoid excessive integration that disrupts cellular functions.

In the Gelderblom et al. study [14], the use of fluorescent reporters had a clear advantage for differentiating various viral and cell populations. An unexpected, striking finding is that although the integrated provirus can chase out many "silent" unintegrated DNA templates, the expression levels from the unintegrated can never match those from the integrated proviruses. There is a clear distinction between these two types of viral DNA templates. Certainly, the possible regulatory mechanism for this difference is of potential interest in the future. Using fluorescent reporters also has its downside: the low sensitivity of fluorescent reporters dictates that a large number of molecules must accumulate in order to be detectable by flow cytometry. This may lead to underestimation of the number of active, unintegrated DNA templates. Some of these "silent" DNA molecules may not be absolutely quiet; instead, it is likely that they actively transcribe, but at a low level "under the radar."

There was also a remote possibility that in the Gelderblom's coinfection experiment, D116N could have integrated with the integrase provided in trans by a coinfecting wild-type virus. However, it is difficult to imagine that the D116N preintegration complex (PIC) could have been disassembled first and then reassembled with a new wild-type PIC. Additionally, during coinfection with the wild-type virus, although the number of active templates was increased, the level of gene expression from D116N was distinctively low, similar to that from the single infection by D116N. This result indicated that the templates were different from the integrated proviral DNA and were likely from the unintegrated.

## Conclusion

Recent years have witnessed an increasing number of studies characterizing unintegrated HIV-1 DNA [9-11,16,17,30,31]. It has become clear that the viral activities from unintegrated DNA are not merely an irrelevant phenomenon distinct from the dominant productive viral replication cycle produced from the integrated proviruses. As demonstrated recently [9,14,16], these two virological processes are intimately intertwined to facilitate viral infection and to overcome certain cellular hurdles. Of course, many fundamental questions remain to be addressed. We still do not understand why most of the viral DNA molecules do not integrate. We also do not know how transcription prior to integration is directly linked with the sequential steps of the viral replication process. Nevertheless, the limited information obtained from basic research on unintegrated DNA does not appear to contradict the recent interest in using unintegrated lentivirus for gene therapy and as attenuated vaccines [32-35]. With additional studies of this biological process, we can look forward to more interesting stories from these "unintegrated."

## Competing interests

The author declares that they have no competing interests.
